# Impact of Antithrombotic Therapy in Atrial Fibrillation on the Presentation of Coronary Artery Disease

**DOI:** 10.1371/journal.pone.0131479

**Published:** 2015-06-22

**Authors:** Pak Hei Chan, Wen Hua Li, Jo Jo Hai, Hung Fat Tse, Chung Wah Siu

**Affiliations:** Cardiology Division, Department of Medicine, Li Ka Shing Faculty of Medicine, the University of Hong Kong, Hong Kong SAR, China; University Hospital Medical Centre, GERMANY

## Abstract

**Background:**

Little is known about whether atrial fibrillation is a presentation of coronary disease. There is a paucity of knowledge about their causal relationship and also the impact of different antithrombotic strategies on the subsequent presentation of symptomatic coronary disease.

**Methods and Results:**

We studied 7,526 Chinese patients diagnosed with non-valvular atrial fibrillation and no documented history of coronary artery disease. The primary endpoint was the new occurrence of coronary artery disease—either stable coronary artery disease or acute coronary syndrome. After a mean follow-up of 3.2±3.5 years (24,071 patient-years), a primary endpoint occurred in 987 patients (13.1%). The overall annual incidence of coronary artery disease was 4.10%/year. No significant differences in age, sex, and mean CHA_2_DS_2_-VASc score were observed between patients with and without the primary endpoint. When stratified according to the antithrombotic strategies applied for stroke prevention, the annual incidence of coronary artery disease was 5.49%/year, 4.45%/year and 2.16%/year respectively in those prescribed no antithrombotic therapy, aspirin, and warfarin. Similar trends were observed in patients with acute coronary syndromes. Diabetes mellitus, smoking history and renal failure requiring dialysis were predictors for primary endpoint in all antithrombotic therapies.

**Conclusion:**

In patients with non-valvular atrial fibrillation, there is a modest association with coronary artery disease. Patients prescribed warfarin had the lowest risk of new onset coronary artery disease.

## Introduction

Atrial fibrillation (AF) is the most common sustained cardiac arrhythmia encountered in clinical practice with prevalence in the general population of approximately 1%.[1] AF is closely linked to coronary artery disease (CAD) as they share some common cardiovascular risk factors, such as advanced age, male gender, hypertension, obesity, and cigarette smoking. In some studies, CAD has been shown to be highly prevalent in patients with AF, affecting 30.5% to 46.5% of individuals.[2–5] This suggests that AF may be a marker of coronary atherosclerosis. Nonetheless such prevalence depends largely on the population studied and the criteria used for diagnosis of CAD, whether it is based on angiographic documentation or on the presence of ECG abnormalities.[6, 7] Conversely, in some subsets of CAD such as post-myocardial infarction patients, the risk of AF recurrence is increased.[8] There is paucity of evidence to explain the causal relationship of AF and CAD despite different postulations of atrial remodelling, ischemia and raised left atrial pressure.[9, 10] Current clinical guidelines do not incorporate specific recommendations on coronary evaluation in patients with AF.[11] Nonetheless for those who present with symptoms mimicking angina, very often, testing for ischemia is performed in order to look for underlying CAD as the possible etiology. It has been suggested that the presence of ST depression during AF, considered to be “positive stress equivalent”, is not consistently associated with the presence of obstructive CAD on angiography or a positive ischemic stress test.[7] Moreover, the impact of antithrombotic therapy for stroke prevention in AF i.e. warfarin or aspirin (as a perceived alternative to warfarin) on the subsequent presentation of CAD in AF patients is largely unknown. In view of the conflicting evidence and paucity of conclusive data depicting the relationship between these two common conditions, this study aims to identify the prevalence of CAD in a large cohort of Chinese patients with AF and the risk factors that predict new CAD occurrence.

## Method

### Study Design and Patients

This study is approved by the Institutional Review Board (IRB) of the University of Hong Kong / Hospital Authority Hong Kong West Cluster. Consent was waived as all the data were analyzed anonymously. This was an observational study based on a hospital-based AF registry.[12–16] Between July 1997 and December 2011, 10,195 Chinese patients with a diagnosis of AF at Queen Mary Hospital, Hong Kong were identified from the computer-based clinical management system. Informed consent was not obtained from patients given the registry nature of the study and that was approved by local IRB as stated; nonetheless, all patient records/information were rendered anonymous prior to analysis.

The index date was defined as the date of the first occurrence of AF. Patients were excluded if they had significant valvular heart disease or any degree of mitral stenosis, previously documented CAD or CAD diagnosed within 14 days of the index date, or incomplete clinical and/or follow-up data. All hospital admissions, outpatient clinic visits, laboratory results and radiological images have been recorded in the computer-based clinical management system since 1996. Demographic data, cardiovascular risk factors, and medications were recorded at baseline.

### Definitions

Hypertension was defined as resting systolic or diastolic blood pressure ≥140/90 mmHg on two occasions or prescription of anti-hypertensive drugs. Diabetes mellitus was defined as a serum fasting glucose ≥7.0mmol/l or prescription of anti-diabetic medication. Significant valvular heart disease included mitral stenosis, any valvular lesions requiring surgery, and previous valvular repair or replacement. Heart failure was defined according to the Framingham Heart Study.[17] The diagnosis of hyperthyroidism was established in the presence of a serum free T4 level > 23 pmol/L, and concomitant suppressed TSH level <0.03 pmol/L.[18–22] Smoking status was recorded as smoker (past and current) or non-smoker. Stroke was defined as a neurological deficit of sudden onset that persisted for more than 24 hours, and corresponded to a vascular territory in the absence of primary hemorrhage, and that could not be explained by other causes (trauma, infection, vasculitis).[23–25]

### Outcomes

The primary endpoint was hospital admission with new occurrence of CAD during the follow-up period, further sub-classified according to clinical presentation into (1) stable CAD, or (2) acute coronary syndrome. Stable CAD was defined as symptoms of angina together with one of the confirmatory tests including computed tomography coronary angiography demonstrating coronary stenosis of any degree, presence of ischemia on myocardial perfusion nuclear scan or magnetic resonance imaging and invasive coronary angiography demonstrating coronary stenosis with or without need for revascularization.[26, 27] Acute coronary syndromes comprised non-ST segment elevation myocardial infarction, and ST-segment elevation myocardial infarction.[28, 29] Data were retrieved from the medical records and discharge summaries from the territory-wide information network of all public hospitals in Hong Kong.

### Statistical Analysis

Continuous and discrete variables are expressed as mean ± standard deviation and percentages, respectively. Statistical comparison of baseline clinical characteristics was performed using Student’s *t* test or one-way ANOVA as appropriate. Kaplan-Meier survival analyses with the log-rank test were carried out and Cox proportional hazards regression model was used to calculate hazard ratios (HRs) of some predictive factors and their 95% confidence interval (CIs) for the incidence of stroke. Calculations were performed using SPSS software (version 21.0). All tests were two-sided, and a *p*-value <0.05 was considered significant.

## Results

**Fig 1:**
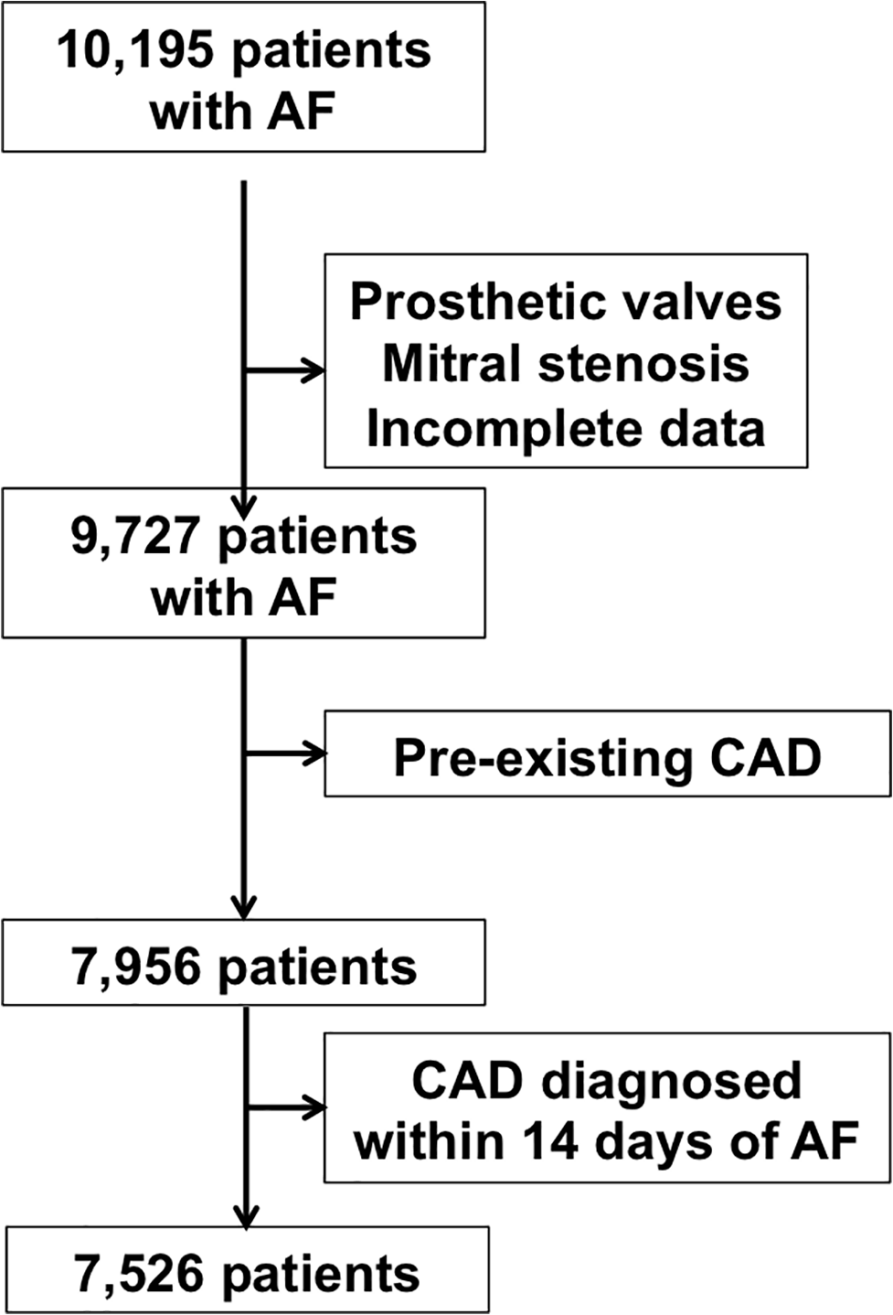
A total of 7,526 Chinese AF patients selected for final analysis.

Of 9,727 patients with non-valvular AF, 1,771 patients with previously diagnosed CAD, and 430 patients with CAD diagnosed within 14 days of the presentation of AF were excluded (Fig 1). The final analysis included 7,526 patients (76.2±13.4 years, female: 56.7%). Table 1 summarizes the clinical characteristics of the study population. There was a high prevalence of hypertension (51.2%), smoker (33.8%), and diabetes mellitus (19%). In addition, 23.3% patients had a history of ischemic stroke. Only 2.8% had a history of peripheral artery disease. Warfarin therapy and aspirin therapy was prescribed in only 18.8% and 36.9% of patients respectively.

**Table 1:** Baseline characteristics of the Study Population.

	All (n = 7,526)	No CAD (n = 6,539)	New CAD (n = 987)	*p-*value
**Age**				
Mean age, (years)	76.2±13.4	76.2±13.8	76.0±10.0	0.72
Female, n (%)	3,909 (51.9)	3,398 (52.0)	511 (51.8)	0.91
Hypertension, n (%)	3,850 (51.2)	3,307 (50.6)	543 (55.0)	<0.01*
Diabetes mellitus, n (%)	1,427 (19.0)	1,187 (18.2)	240 (24.3)	<0.0001*
Smoker, n (%)	2,545 (33.8)	2,168 (33.2)	337 (38.2)	<0.01*
Hyperthyroidism, n (%)	535 (7.1)	470 (7.2)	65 (6.6)	0.49
Renal failure on dialysis, n (%)	125 (1.7)	92 (1.4)	33 (3.3)	<0.0001*
Heart failure, n (%)	1,442 (19.2)	1,264 (19.3)	178 (18.0)	0.34
Peripheral arterial disease, n (%)	211 (2.8)	184 (2.8)	27 (2.7)	0.89
Ischemic stroke/TIA, n (%)	1,751 (23.3)	1,574 (24.1)	177 (17.9)	<0.0001*
Intracranial hemorrhage, n (%)	182 (2.4)	170 (2.6)	12 (1.2)	<0.01*
Mean CHA_2_DS_2_-VASc score	3.4±1.7	3.4±1.7	3.3±1.6	0.34
**Antithrombotic therapy**				
No therapy, n (%)	3,330 (44.3)	2,896 (44.3)	434 (44.0)	0.01*
Aspirin, n (%)	2,780 (36.9)	2,383 (36.4)	397 (40.2)	
Warfarin, n (%)	1,416 (18.8)	1,260 (19.3)	156 (15.8)	

*p<0.05.

### Coronary artery disease

After a mean follow-up of 3.2±3.5 years (24,071 patient-years), 987 patients had a new occurrence of CAD (13.1%). The overall annual incidence of coronary artery disease was 4.10%/year. There was no significant difference in age, gender, and mean CHA_2_DS_2_-VASc score between patients with and without new occurrence of coronary artery disease (Table 1). Nonetheless patients with new occurrence coronary artery disease had a higher prevalence of hypertension (55.0% *vs*. 50.6%, *p*<0.01), diabetes mellitus (24.3% *vs*. 18.2%, *p*<0.0001), smoking history (38.2% *vs*. 33.2%, *p*<0.01), and renal failure on dialysis (3.3% *vs*.1.4%, *p*<0.0001) than those without new occurrence of coronary artery disease. Interestingly, patients with new occurrence of coronary artery disease had a lower prevalence of both prior ischemic stroke (17.9% *vs*. 24.1%, *p*<0.0001) and previous intracranial hemorrhage (2.6% *vs*. 1.2%, *p*<0.01).

**Fig 2:**
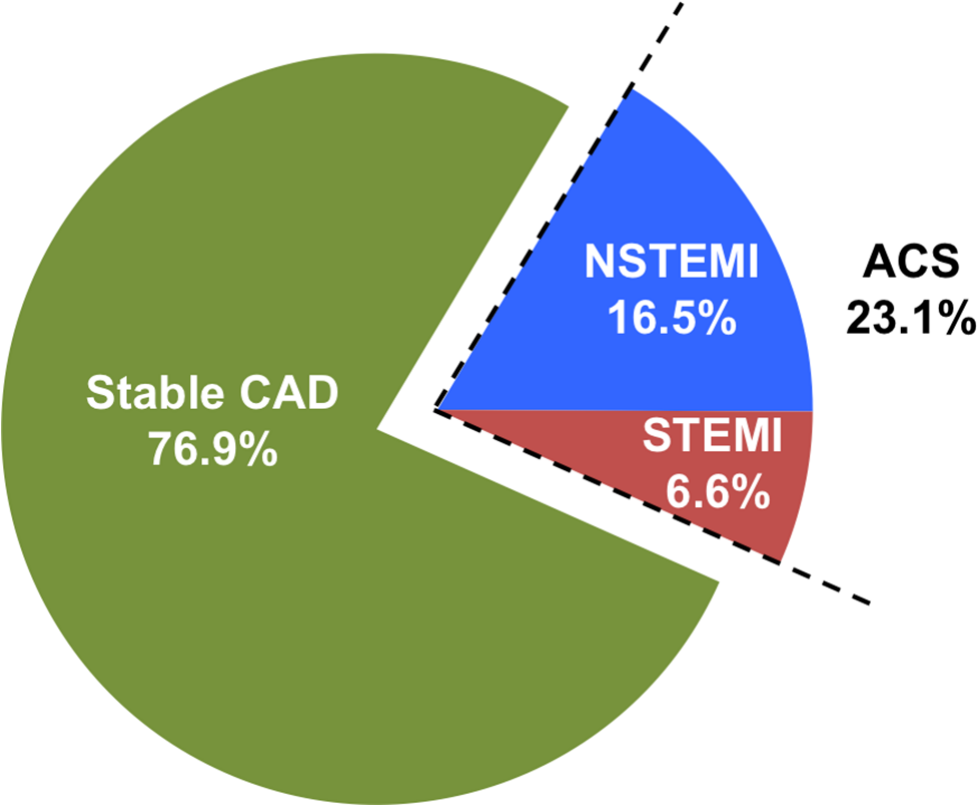
Proportion of patients presenting with stable CAD and ACS (both NSTEMI and STEMI) amongst new occurrence of CAD.

**Fig 3:**
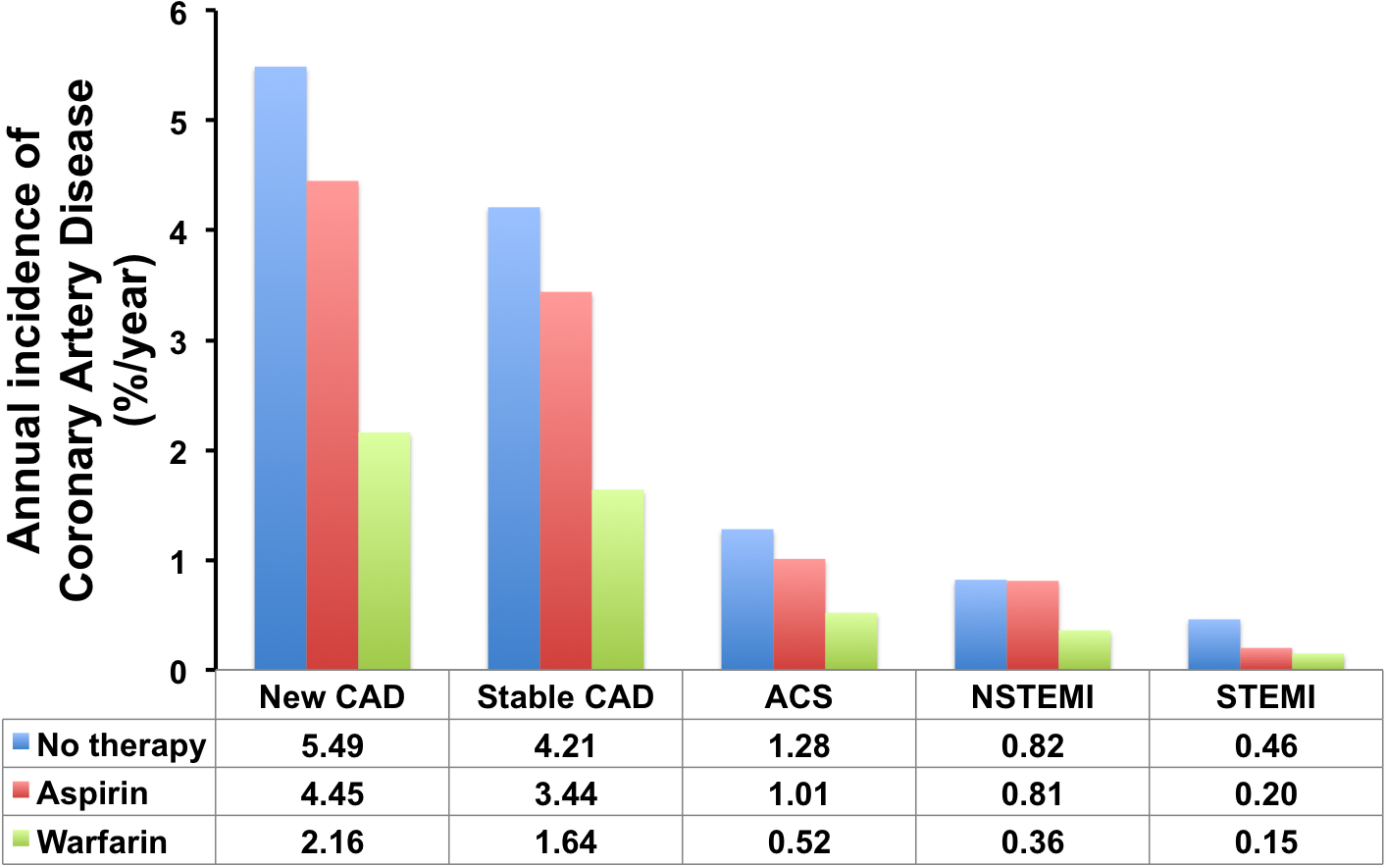
Annual incidence of new occurrence of CAD with different presentations comparing amongst different antithrombotic therapy for stroke prevention in AF; warfarin is consistently associated with lowest incidence in all CAD presentations.

Amongst those with new occurrence of CAD, 759 patients (76.9%) presented with stable CAD and the remaining 228 patients (23.1%) presented with acute coronary syndrome with either non-ST elevation myocardial infarction (16.5%) or ST-elevation myocardial infarction (6.6%)(Fig 2). The annual incidence of stable CAD was 3.15%/year whereas the annual incidence of acute coronary syndrome was 0.95%/year (non-ST elevation myocardial infarction: 0.7%/year and ST-elevation myocardial infarction: 0.27%/year). When stratified according to antithrombotic therapy, the annual incidence of CAD was 5.49%/year, 4.45%/year and 2.16%/year in those prescribed no antithrombotic therapy, aspirin therapy, and warfarin therapy respectively. Similar trends were likewise observed in acute coronary syndromes (1.3%/year in patients with no antithrombotic therapy, 1.0%/year for aspirin therapy, and 0.5%/year for warfarin therapy)(Fig 3).

### Predictors for new occurrence of coronary artery disease

Table 2 summarizes factors predictive of new occurrence of CAD together with the corresponding HRs based on Cox proportional hazard model and 95% CIs in patients with no antithrombotic therapy, aspirin therapy and warfarin therapy. Across these three antithrombotic strategies, diabetes mellitus, smoking history, and renal failure requiring dialysis were consistently associated with an increased risk of new occurrence of CAD in both univariate and multivariate analysis. In addition, amongst patients prescribed no therapy and aspirin therapy, hypertension was likewise associated with an increased risk of new occurrence of CAD; previous ischemic stroke was paradoxically associated with a lower risk of new occurrence of CAD in these two groups of patients. In addition, in patients prescribed warfarin, age ≥75 years and heart failure at baseline were two additional risk factors associated with new onset CAD. Table 3 summarizes factors predictive of new occurrence of CAD in the whole cohort of AF patients.

**Table 2:** Association between baseline factors and new onset CAD, stratified by different antithrombotic therapies.

	Number of new coronary artery disease	Univariate Analysis		Multivariate Analysis	
		HR (95% CI)	*P*-value	HR (95% CI)	*P*-value
**Patients prescribed no antithrombotic therapy (n = 3,330)**					
Age ≥75 years	257	1.23 (1.01–1.50)	0.04*	1.13 (0.93–1.38)	0.22
Female	234	0.98 (0.81–1.18)	0.83		
Hypertension	218	1.67 (1.38–2.01)	<0.0001*	1.47 (1.20–1.80)	<0.0001*
Diabetes mellitus	99	1.71 (1.37–2.15)	<0.0001*	1.46 (1.15–1.85)	<0.0001*
Smoker	179	1.36 (1.13–1.65)	0.001	1.44 (1.20–1.74)	<0.0001*
Hyperthyroidism	30	0.68 (0.47–0.99)	0.05*	0.72 (0.50–1.04)	0.08
Renal failure on dialysis	16	2.30 (1.39–3.79)	0.001*	2.03 (1.22–3.38)	<0.01*
Heart failure	68	1.50 (1.15–1.94)	0.002	1.30 (1.00–1.69)	0.05
Peripheral arterial disease	6	2.27 (1.01–5.08)	0.05*	1.50 (0.67–3.40)	0.33
Prior ischemic stroke	61	1.11 (0.84–1.45)	0.46		
Prior Intracranial hemorrhage	6	0.55 (0.24–1.22)	0.14		
**Patients prescribed aspirin therapy (n = 2,780)**					
Age ≥75 years	236	0.90 (0.74–1.10)	0.31		
Female	200	0.85 (0.69–1.03)	0.09	0.93 (0.75–1.51)	0.49
Hypertension	236	1.32 (1.08–1.61)	<0.01*	1.26 (1.02–1.56)	0.03*
Diabetes mellitus	103	1.48 (1.18–1.85)	0.001*	1.36 (1.08–1.72)	0.01*
Smoker	145	1.25 (1.02–1.53)	0.03*	1.25 (1.00–1.56)	0.06
Hyperthyroidism	23	0.70 (0.46–1.06)	0.09	0.73 (0.48–1.12)	0.15
Renal failure on dialysis	12	2.01 (1.13–3.58)	0.02*	1.85 (1.04–3.30)	0.04*
Heart failure	76	0.96 (0.75–1.24)	0.74		
Peripheral arterial disease	13	1.02 (0.59–1.77)	0.95		
Prior ischemic stroke	76	0.73 (0.57–0.94)	0.02*	0.71 (0.55–0.91)	<0.01*
Prior Intracranial hemorrhage	4	0.60 (0.22–1.60)	0.31		

*p<0.05.

**Table 3:** Associations between baseline factors and new onset coronary artery disease (n = 7,526).

	Number of new coronary artery disease	Univariate Analysis		Multivariate Analysis	
		HR (95% CI)	*P*-value	HR (95% CI)	*P*-value
**Age**					
<75	423	Reference		Reference	
≥75	564	1.24 (1.09–1.41)	0.001*	1.09 (0.85–1.24)	0.21
Female	511	1.00 (0.88–1.13)	0.94		
Hypertension	543	1.46 (1.29–1.66)	<0.0001*	1.39 (1.21–1.59)	<0.0001*
Diabetes mellitus	240	1.63 (1.41–1.89)	<0.0001*	1.44 (1.24–1.68)	<0.0001*
Smoker	377	1.27 (1.11–1.44)	<0.0001*	1.32 (1.16–1.50)	<0.0001*
Hyperthyroidism	65	0.72 (0.56–0.92)	0.01*	0.74 (0.57–0.95)	0.02*
Renal failure on dialysis	33	2.44 (1.72–3.46)	<0.0001*	2.08 (1.46–2.96)	<0.0001*
Heart failure	178	1.22 (1.04–1.44)	0.02*	1.13 (0.96–1.34)	0.15
Peripheral arterial disease	27	1.11 (0.76–1.64)	0.57		
Prior ischemic stroke	177	0.85 (0.72–1.00)	0.05	0.85 (0.72–1.01)	0.06
Prior Intracranial hemorrhage	12	0.64 (0.36–1.12)	0.12		
**Antithrombotic therapy**					
No antithrombotic, n (%)		Reference		Reference	
Aspirin, n (%)		0.89 (0.78–1.02)	0.11	0.84 (0.73–0.96)	0.01*
Warfarin, n (%)		0.44 (0.36–0.52)	<0.0001*	0.43 (0.36–0.52)	<0.0001*

*p<0.05

### Impact of different antithrombotic therapies on new occurrence of coronary artery disease

To allow better understanding on the impact of different antithrombotic therapies on the incidence of coronary artery disease in AF patients, baseline characteristics of 3 different groups of patients, those on aspirin, warfarin and those not on antithrombotic therapy, were compared. Table 4 shows the difference in baseline characteristics of patients receiving different antithrombotic therapies. Of note, patients on warfarin were associated with lowest incidence of new occurrence of CAD, relatively younger, of male predominance (52.9%), and having higher incidence of peripheral artery disease and prior ischemic stroke or TIA compared to other 2 groups. The mean CHA_2_DS_2_-VASc scores were 3.2±1.6, 3.7±1.7 and 3.3±1.8 for patients receiving no therapy, aspirin and warfarin respectively.

**Table 4:** Baseline characteristics of the patients with no therapy, aspirin, and warfarin.

	No therapy (n = 3,300)	Aspirin (n = 2,780)	Warfarin (n = 1,416)	*p-*value
**Age**				
Mean age, (years)	76.7±13.9	77.7±12.2	71.8±13.3	<0.01*
Female, n (%)	1,769 (53.1)	1,473 (53.0)	667 (47.1)	<0.01*
Hypertension, n (%)	1,518 (45.6)	1,609 (57.9)	723 (51.1)	<0.01*
Diabetes mellitus, n (%)	556 (16.7)	590 (21.2)	281 (19.8)	<0.01*
Smoker, n (%)	1,143 (34.3)	949 (34.1)	453 (32.0)	0.27
Hyperthyroidism, n (%)	231 (6.9)	192 (6.9)	112 (7.9)	0.43
Renal failure on dialysis, n (%)	55 (1.7)	48 (1.7)	22 (1.6)	0.92
Heart failure, n (%)	550 (16.5)	608 (21.9)	284 (20.1)	<0.01*
Peripheral arterial disease, n (%)	38 (1.1)	100 (3.6)	73 (5.2)	<0.01*
Ischemic stroke/TIA, n (%)	582 (17.5)	768 (27.6)	401 (28.3)	<0.01*
Intracranial hemorrhage, n (%)	95 (2.9)	59 (2.1)	28 (2.0)	0.09
Mean CHA_2_DS_2_-VASc score	3.2±1.6	3.7±1.7	3.3±1.8	<0.01*

*p<0.05

## Discussion

In this study, we described the incidence, time course, and clinical predictors of new occurrence of CAD in patients who first presented with AF. Our results showed that patients with AF and no previously documented CAD were at moderate risk of new occurrence of coronary artery disease with an overall annual incidence of 4.10%. Although the majority of patients with new occurrence of CAD presented as stable CAD, around a quarter of these CAD patients presented with acute coronary events. Traditional cardiovascular risk factors such as hypertension, diabetes mellitus, smoking history, and renal failure requiring dialysis predicted the occurrence of new CAD. Many of these cardiovascular risk factors are also risk factors for AF development. [30] Owing to this special link between these two common cardiovascular conditions, efforts have been made to evaluate their pathophysiological mechanism and causal relationship. In patients with CAD, previous studies have demonstrated that those with transient AF during myocardial infarction are at higher risk of subsequent recurrence of AF and also higher risk of ischemic stroke. [8, 31] Nonetheless due to the observational nature of these studies, the best clinical approach to manage this group of patients remains undecided. Conversely, for patients with newly diagnosed AF, cross-sectional studies have demonstrated a high prevalence of CAD, ranging from 30.5% to 46.5%,[2–5] As pointed out earlier though, due to the heterogeneity in diagnostic methods for CAD, different populations studied and also the associated background cardiovascular risk factors, the true correlation between these two conditions may not be reflected. One study examined a cohort of 2768 patients with AF and mean age of 71 years.[32] Seventeen percent of these patients developed a first coronary event after a mean follow up of 6.0 years, giving an annual incidence of 3.1%/year, similar to our reported 4.1%/year. In that particular study,[32] the majority of coronary ischemic events were due to acute coronary events or sudden cardiac death occurring within the first year of diagnosis of AF. Only 32% of men and 19% of women were categorised as having stable CAD.

In our study, stable CAD accounted for the majority (76.9%) of clinical presentations and the annual incidence was 3.15%/year. When stratified according to antithrombotic therapy for AF, the annual incidence in patients receiving warfarin was the lowest (2.16%/year). Those prescribed no therapy had the highest incidence (5.49%/year) and aspirin users had an incidence of 4.45%/year. A similar trend was also observed for patients with AF who subsequently presented with new acute coronary syndrome. In our cohort, those patients prescribed warfarin as antithrombotic strategy and to a lesser extent those prescribed aspirin for stroke prevention appeared to have a lower risk of both stable CAD and acute coronary events.

Of note, across these three antithrombotic management strategies, diabetes mellitus, smoking history, and renal failure requiring dialysis were consistently associated with an increased risk of new occurrence of CAD. Likewise, hypertension was associated with an increased risk in those patients not prescribed warfarin. Although it is difficult to draw a conclusion from this observation that antithrombotic therapy reduced subsequent coronary events—both stable CAD and acute coronary syndrome, there may be certain treatment implications. To provide insights on impact of different antithrombotic therapy on incidence of coronary events, baseline patient characteristics were compared between the three groups. Despite having lowest new occurrence of CAD among patients on warfarin, they were of similar baseline risk compared with the other two groups, with similar CHA_2_DS_2_-VASc score and paradoxically associated with higher incidence of prior ischemic stroke and peripheral artery disease compared with patients on aspirin or no therapy. In prior studies comparing aspirin and warfarin as secondary prevention for subsequent coronary events, warfarin was shown to be of similar efficacy compared with aspirin.[33, 34] In a randomized trial comparing aspirin and warfarin for primary prevention of coronary events,[35] it was shown that absolute reductions in all coronary events due to warfarin or aspirin were 2.6 and 2.3 per 1000 person years respectively. This demonstrates the efficacy of warfarin in reducing subsequent coronary events, which possibly explain the lower incidence of symptomatic CAD presentation for those taking warfarin, as well as aspirin compared with no therapy.

**Fig 4:**
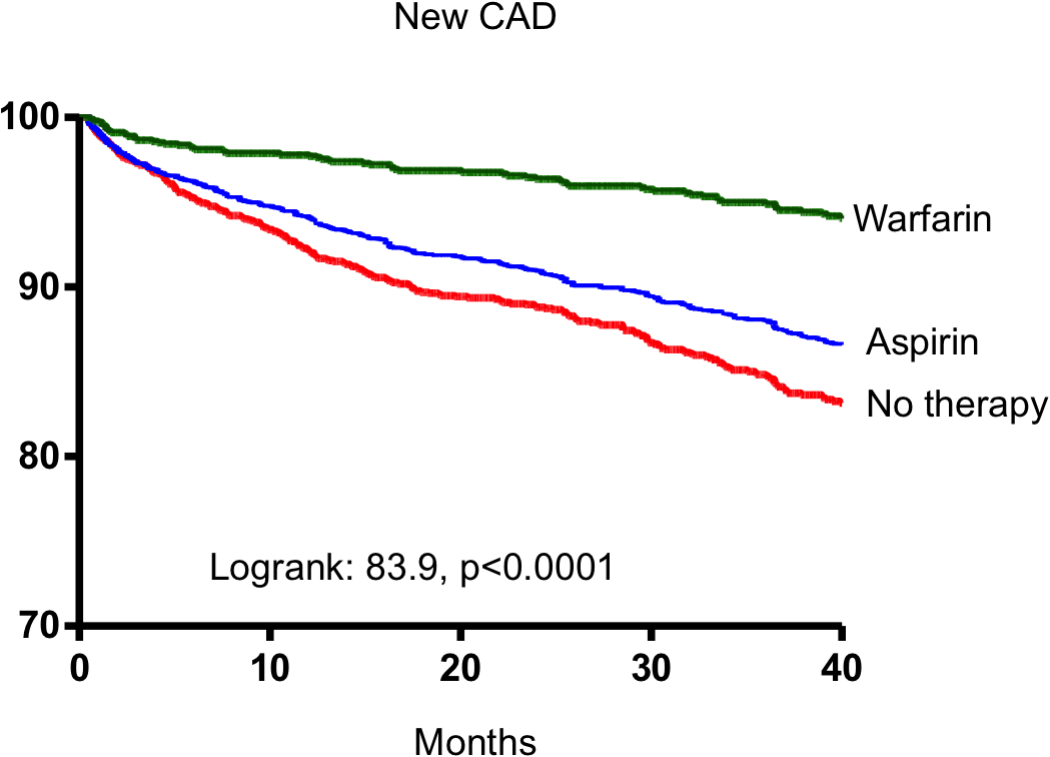
Kaplan-Meier estimates of all incident CAD-free survival in AF patients receiving warfarin, aspirin and no therapy.

**Fig 5:**
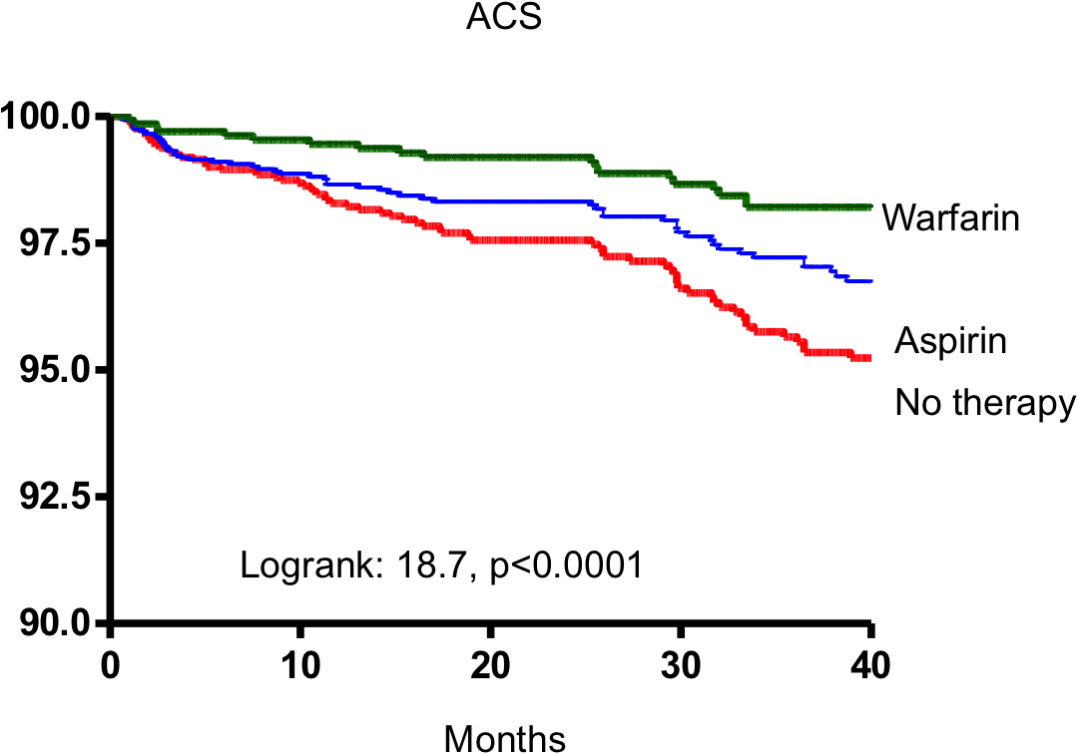
Kaplan-Meier estimates of all ACS-free survival in AF patients receiving warfarin, aspirin and no therapy.

Current clinical guidelines recommend antithrombotic therapy in patients with AF when CHA_2_DS_2_-VASc ≥1.[11, 36] From this study, common risk factors such as diabetes mellitus and hypertension, components of CHA_2_DS_2_-VASc scoring 1 mark, were associated with increased risk of new occurrence CAD. The presence of either of these risk factors will confer an increased risk of ischemic stroke and thus indicate the need for antithrombotic therapy. This study suggested that prescription of antithrombotic therapy in this group of patients will not only lower the subsequent risk of ischemic stroke,[13] but might also lower the risk of new occurrence of CAD, both as stable angina and acute coronary syndromes. (Figs 4 and **5**).

In view of the perceived association between CAD and AF, patients who present with AF without angina might still be investigated for possible underlying CAD. It has been demonstrated that the presence of ST depression during the initial presentation of AF might be associated with a higher prevalence of CAD and vice versa. A high negative predictive value for CAD in patients without ST depression on ECG has also been observed.[37] This signifies the cost-effectiveness of evaluation for CAD only in the presence of ST depression.

With regard to diagnostic tests, in patients with multiple cardiovascular risk factors and high pre-test probability for CAD, such as those with typical angina, invasive coronary angiography is advisable.[26] Alternatively, non-invasive tests can be considered. Coronary computed tomography angiography (CCTA), thallium myocardial perfusion scintigraphy (MPS) and dobutamine stress echocardiography have been used to evaluate this particular subset of patients. It should be noted though that MPS was shown to be of low specificity (58.9%) and positive predictive value (50%) in one study.[7]

For patients who present with AF and who have multiple cardiovascular risk factors, a more aggressive workup may allow early detection and thus prompt management of coronary artery disease. Evidence for the optimal threshold for CAD evaluation and appropriate investigation(s) of choice are still lacking. In our study, the subsequent event rate for new occurrence of stable CAD and acute coronary syndromes in patients on antithrombotic therapy with warfarin were 2.16%/year and 0.5%/year respectively, both of which were low. Whether earlier testing for CAD followed by standard appropriate therapy for CAD will further improve prognosis is largely unknown. A large-scale randomized controlled trial in specific subsets of patients with a high prevalence of cardiovascular risk factors might be warranted in order to determine the efficacy of routine CAD evaluation.

### Limitations

This study was limited by its registry-based and single-center observational design in primarily hospital-based patients. AF patients in this cohort might have a different cardiovascular risk profile to those with AF seen only in an out-patient setting and with no acute symptoms. Data on cholesterol level at diagnosis of AF and use of a statin as an important part of medical therapy for CAD were also lacking in our current study. Due to the observational nature of our study, asymptomatic CAD was not routinely screened for and therefore the true incidence of CAD at diagnosis of AF was not known. In addition, the decision to prescribe no therapy, aspirin and warfarin was based on the attending physician taking into consideration patient’s baseline clinical characteristics, which might partly explain relatively large proportion of patients receiving either no antithrombotic or aspirin. Finally, although we controlled the potential confounders using multivariate logistic regression, the use of a case-control design to compare patients with and without AF may also be appropriate.

## Conclusion

In patients with non-valvular atrial fibrillation, there is a modest association with coronary artery disease with lowest risk of among those who take warfarin. There is a paucity of evidence to support a routine evaluation for coronary artery disease in patients with newly diagnosed atrial fibrillation due to the low incidence.
